# No Effect of Featural Attention on Body Size Aftereffects

**DOI:** 10.3389/fpsyg.2016.01223

**Published:** 2016-08-22

**Authors:** Ian D. Stephen, Chloe Bickersteth, Jonathan Mond, Richard J. Stevenson, Kevin R. Brooks

**Affiliations:** ^1^Department of Psychology, Macquarie University, Sydney, NSWAustralia; ^2^ARC Centre of Excellence in Cognition and its Disorders, Macquarie University, Sydney, NSWAustralia; ^3^Perception in Action Research Centre, Faculty of Human Sciences, Macquarie University, Sydney, NSWAustralia; ^4^Centre for Health Research, School of Medicine, Western Sydney University, Campbelltown, NSWAustralia

**Keywords:** body perception, adaptation aftereffects, featural attention, body fatness, body size misperception

## Abstract

Prolonged exposure to images of narrow bodies has been shown to induce a perceptual aftereffect, such that observers’ point of subjective normality (PSN) for bodies shifts toward narrower bodies. The converse effect is shown for adaptation to wide bodies. In low-level stimuli, object attention (attention directed to the object) and spatial attention (attention directed to the location of the object) have been shown to increase the magnitude of visual aftereffects, while object-based attention enhances the adaptation effect in faces. It is not known whether featural attention (attention directed to a specific aspect of the object) affects the magnitude of adaptation effects in body stimuli. Here, we manipulate the attention of Caucasian observers to different featural information in body images, by asking them to rate the fatness or sex typicality of male and female bodies manipulated to appear fatter or thinner than average. PSNs for body fatness were taken at baseline and after adaptation, and a change in PSN (ΔPSN) was calculated. A body size adaptation effect was found, with observers who viewed fat bodies showing an increased PSN, and those exposed to thin bodies showing a reduced PSN. However, manipulations of featural attention to body fatness or sex typicality produced equivalent results, suggesting that featural attention may not affect the strength of the body size aftereffect.

## Introduction

Body size misperception is the perceptual effect wherein a person’s view of their body size is inaccurate. That is, they view themselves as larger or smaller than they really are. This has implications for people who fail to recognize that they are overweight, and thus are less likely to take steps to lose weight, increasing their risk of diabetes and hypertension (Powell et al., 2010). Body size misperception is also associated with *anorexia nervosa* (Stice, 2002), body dissatisfaction, negative affect, eating-disordered behavior and poor mental health (Stice et al., 2003; Paxton et al., 2006) in underweight and normal weight people who perceive themselves to be overweight (McCreary et al., 2004).

Body misperception is often attributed to exposure to unrealistic body ideals (thin for women, lean and muscular for men; Furnham et al., 2002), such as those presented in the mass media. Malkin et al. (1999) found that 94% of covers in a survey of 69 American women’s magazines featured a thin-idealized subject. Research has found that exposing women to extremely thin or fat bodies significantly alters their perception of body normality and ideals (Glauert et al., 2009). This is supported by other studies demonstrating that both long-term and short-term exposure to idealized stimuli (i.e., thin bodies for women and muscular bodies for men) is associated with negative body image in women (Groesz et al., 2002) and men (Barlett et al., 2008). More recent evidence suggests that exposure to such images can affect observers’ perceptions of what makes an attractive female body (Stephen and Perera, 2014).

Whilst the effects that occur after exposure to these images have been repeatedly demonstrated, little is known about the perceptual mechanisms behind body misperception. Recently, studies under controlled laboratory conditions have shown a causal link between exposure to thin bodies and body size misperception, implicating a visual adaptation effect in the process (Winkler and Rhodes, 2005; Glauert et al., 2009; Brooks et al., 2015; Sturman et al., submitted). Adaptation is the well-studied perceptual phenomenon whereby prolonged exposure to an “extreme” visual stimulus leads to an aftereffect such that a “normal” stimulus appears distorted in the other direction. For example, in motion perception, exposure to downward motion results in a subsequently viewed stationary scene appearing to drift upward (Addams, 1834). In color perception, adapting to the color green results in neutral stimuli being perceived as red because red and green are perceptually opposed in the human visual system (Padgham, 1953; Hurvich and Jameson, 1957). These are examples of low-level aftereffects, as they involve simple stimulus attributes that are known to be processed early in the visual system.

However, in recent research the adaptation phenomenon has been demonstrated in higher-level properties of stimuli, including the gender (Webster and MacLeod, 2011; Hummel et al., 2012b), race (Webster et al., 2004), identity (Rhodes et al., 2013), and geometric structure (Gwinn and Brooks, 2013, 2015a,b) of faces. These face aftereffects are found even when the adaptation and test stimuli are presented at different locations on the screen, or at different sizes, indicating that they are not retinotopic, as would be expected of low-level effects (Leopold et al., 2005; Rhodes et al., 2011).

Aftereffects are also found in body stimuli, including adaptation to higher-level percepts such as gender (Palumbo et al., 2013, 2014). Many studies finding that after exposure to thin or fat bodies, perceptions of body normality and ideals are significantly changed (Winkler and Rhodes, 2005; Glauert et al., 2009; Hummel et al., 2012a,b). For example, observers who adapted to a picture of their own body that had been manipulated to appear thinner perceived a subsequently presented picture of their undistorted body as being fatter than it actually was, and vice versa (Hummel et al., 2012b). Body size aftereffects cannot be induced by exposure to wide or narrow rectangles (Hummel et al., 2012a), suggesting that, as for faces, body size aftereffects are attributable to adaptation of higher level brain mechanisms, rather than to simple mechanisms earlier in the visual system representing stimulus shape.

While humans in a given geographical area are exposed to approximately the same ‘set’ of bodies in the environment, not all individuals suffer from body size misperception. The reasons for this difference in susceptibility have yet to be established. One possible candidate may be the degree of attention that is paid to the environmental stimuli, with some individuals selectively attending to the size of bodies more than others. More recently, it has been shown that, while eating disordered patients show normal adaptation aftereffects following exposure to images of fat bodies, they do not show the expected aftereffects following exposure to images of thin bodies, suggesting that “pre-adaptation” to thin bodies may be a characteristic of eating disorders (Mohr et al., 2016).

Research suggests that increased attention to a particular stimulus increases the neural responses to that stimulus (Pestilli et al., 2007). This increased neural activity is accompanied by increased levels of neural adaptation, as shown by demonstrations that the motion aftereffect (Rezec et al., 2004), the figural aftereffect (Yeh et al., 1996), and the direct tilt aftereffect (Spivey and Spirn, 2000) are all increased by spatial attention (attention directed to the location of the stimulus). Object attention (attention directed to a particular object within a space) has also been shown to enhance the direct tilt aftereffect (Spivey and Spirn, 2000), and the figural aftereffect (Shulman, 1992). Similarly, featural attention has been found to increase the strength of the motion aftereffect (Lankheet and Verstraten, 1995; Boynton et al., 2006).

In faces, increased object attention enhances the strength of the identity adaptation effect (Rhodes et al., 2011), suggesting that other high-level aftereffects may also be susceptible to enhancement by attention. However, a recent report in the current volume shows that featural attention to the ethnicity or gender of faces does not impact the strength of aftereffects along ethnicity and gender dimensions (Davidenko et al., 2016). Yet little is known about the impact of featural attention to body fatness on body size aftereffects.

Here, we assess the impact of featural attention on the body size adaptation effect. All observers will see the same adaptation stimuli, but in the fatness attention condition they will rate the bodies according to their adiposity, whereas in the sex typicality attention condition observers will rate the bodies according to their sex typicality. We predict that selective attentional bias (i.e., attending different featural aspects of bodies) will modulate the magnitude of body size aftereffects. Observers engaging in a task explicitly rating body fatness are predicted to experience stronger body size adaptation effects than those who rate the same bodies for a non-fatness attribute (sex typicality).

Previous studies on the body size adaptation effect have tended to use stimuli that are either computer generated bodies (Glauert et al., 2009), or used simple width (Winkler and Rhodes, 2005) or simulated surface area (Hummel et al., 2012a) as a proxy for body fatness. Here, we use photographs of real people, manipulated along an empirically measured body fat axis to enhance ecological validity.

## Materials and Methods

All work was approved by the Macquarie University Human Research Ethics Committee. All observers gave prior informed consent in writing.

### Participants

Throughout this paper, we refer to participants who were photographed for the stimuli as “subjects,” and to participants who give perceptual responses as “observers.” One hundred and ninety two Caucasian subjects aged 18–30 (*M* = 20.76, *SD* = 5.35) were recruited (128 females, 64 males). Subjects received course credit or $20 for their time. Eighty-nine Caucasian observers, aged 18 and 29 (*M* = 21.4, *SD* = 3.02) were recruited (45 males, 46 females). Observers were compensated for their time with course credit or $10.

### Design

This experiment used a 2 × 2 × 2 between-subjects design, with three independent variables: attention (fatness or sex typicality), adiposity of adaptation stimuli (high or low fat) and sex. Male observers saw only male images and female observers saw only female images, as we are primarily interested in the adaptation to own sex stimuli. The dependent variable was change in the Point of Subjective Normality (ΔPSN) from baseline to adaptation testing. PSN was measured using a method of adjustment task that allowed observers to manipulate the adiposity of a body to make them look as normal as possible.

### Stimuli and Apparatus

Full body photographs of the subjects were taken and used to create the stimuli. Subjects wore a standard gray tight fitting singlet and gray tight fitting shorts provided by the researcher. They were asked to pull their hair back, to remove all jewelry and skin make-up, and to pose in anatomical position (upright, legs shoulder width apart, facing the camera with arms by their side, palm facing forward) with a neutral facial expression. Markings on the floor were used to ensure a standardized position for all subjects.

Photographs were taken in a light booth painted Munsell N5 neutral gray and illuminated with 15 Verivide F20 T12/D65 daylight simulation bulbs mounted in high-frequency fittings to reduce the effects of flicker (Verivide, UK; Stephen et al., 2011). No other light source was present in the room. Photographs were taken using a Canon D50 digital SLR camera with the settings held constant.

**FIGURE 1:**
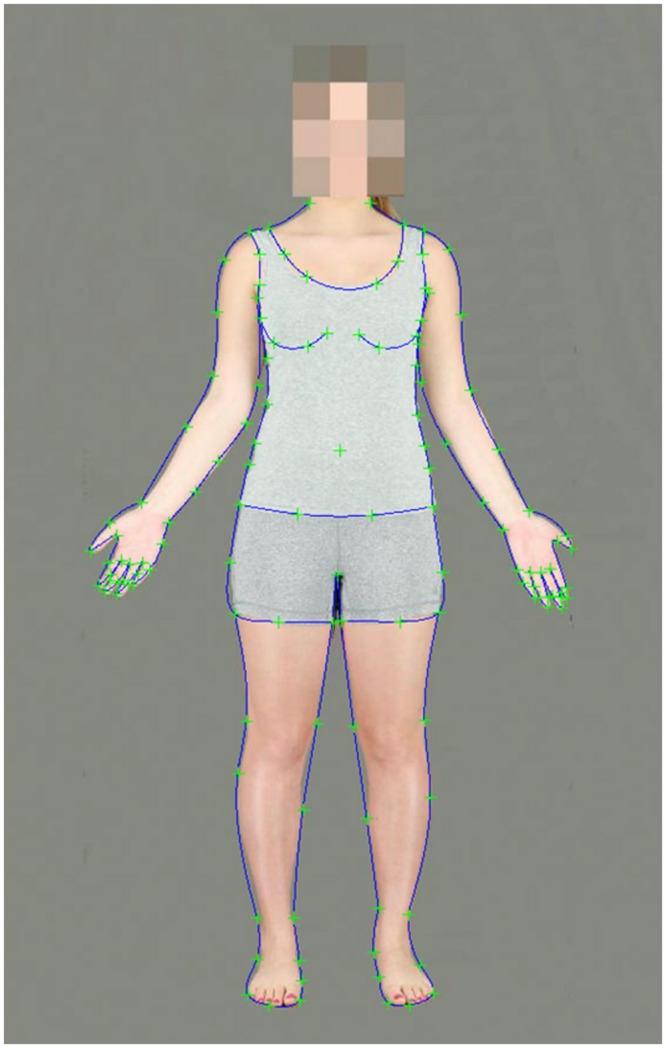
**A female body image delineated with 130 landmark points**.

A Tanita SC 330 body composition analyser was used to accurately measure body fat and muscle mass. Height was measured manually using a fixed measuring tape. One hundred and thirty landmark points were delineated on the body images using Psychomorph (Tiddeman et al., 2001; **Figure 1**).

Since body fat mass is correlated with muscle mass and height, linear regression was used to estimate the fat mass of the subjects, controlling for muscle mass and height, following Brierley et al. (2016).

The 10 female bodies with the highest and the 10 female bodies with the lowest fat mass (controlling for muscle mass and height) were selected. Independent sample *t*-tests showed that these two groups differed significantly in fat mass [*M*_diff_ = 12kg; *t*(18) = 4.10; *p* = 0.001], but not muscle mass [*M*_diff_ = 1.5kg; *t*(18) = 0.96; *p* = 0.350] or height [*M*_diff_ = 2.27 cm; *t*(18) = 0.94; *p* = 0.362].

The 10 female bodies with the highest fat mass (controlling for muscle mass and height) were used to produce a high fat composite image in PsychoMorph. This procedure involved finding the mean coordinates for each landmark across the 10 subjects to form an average shaped template. Each subject image was then warped into the shape of the average template, and the mean color was calculated across the 10 subject images at each pixel to form an average high fat mass residual female image. A similar procedure was used to create an average low fat mass residual female image from the 10 lowest fat mass residual subject images. These average images formed endpoints for the transformations.

All images were aligned in PsychoMorph to remove variation in translation and rotation. They were also resized to 600 × 900 pixels. The background of all images was changed to be a uniform gray using Photoshop.

**FIGURE 2:**
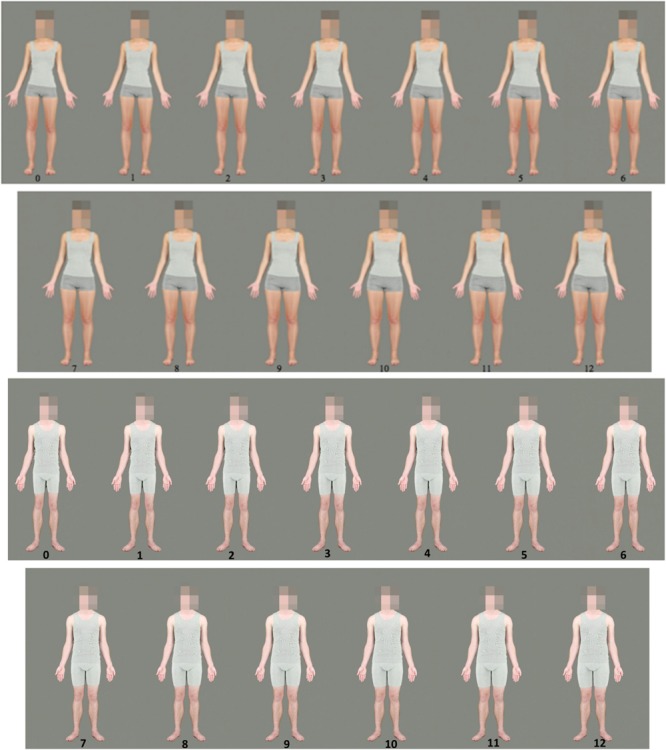
**An example of a transformed female body (top) and male body (bottom).** Frame 0 has been reduced by 12 kg of fat, Frame 12 has been increased by 12 kg. Each step represents a change of 2 kg fat mass.

Following Stephen and Perera (2014), 50 female subject images were then transformed in PsychoMorph according to the difference between the two endpoint composite images. This was achieved by calculating the difference in location between the two endpoint images for each landmark point to form a vector. For each subject image, each landmark point was moved along the corresponding vector to simulate increased or decreased fat mass. These transformations were conducted in 13 equidistant steps. The smallest body image was thinner than the original by 100% of the difference between the endpoint images (-12 kg), while the largest was fatter by 100% of the difference between the endpoint images (+12 kg). The middle image was the unmanipulated original photograph, and each step represented a change of 2 kg fat mass (see **Figure 2**).

This process created images that retained the identity cues of the original images, but varied in terms of apparent body fat composition. Faces were blurred using the Photoshop pixelate mosaic function (which averaged pixel color over a square 45 × 45 pixels in size), to render individuals’ faces unidentifiable and to obscure facial shape changes introduced by the transforms.

The process was then repeated for the 10 highest and 10 lowest fat male images (controlling for muscle mass and height). Independent samples *t*-tests from the male data showed that these two groups differed significantly in fat mass [*M*_diff_ = 12 kg; *t*(18) = 2.48; *p* = 0.023], but not muscle mass [*M*_diff_ = 0.04 kg; *t*(18) = 0.01; *p* = 0.995] or height [*M*_diff_ = -2.21 cm; *t*(18) = 0.61; *p* = 0.548]. The transformations were conducted on 50 male identities in the same way as the female images.

Ten male and 10 female subject identities were randomly selected to be used in the PSN method of adjustment tasks, with the remaining identities to be piloted for use as adaptation stimuli.

#### Pilot

For the 40 remaining male and 40 remaining female identities, seven of the 13 frames per identity (excluding every other image in the sequence) were used in a pilot study. Observers were asked to rate how fat each body appears, using the full range of a 7-point Likert scale ranging from very thin to very fat. Ten Caucasian males (aged 18–30) completed a pilot of male images and 10 Caucasian females (aged 18–30) completed a pilot of female images. Each observer therefore rated 280 (7 frames × 40 identities, presented in random order) of his/her own gender.

GraphPad Prism (Version 6; 2015) was used to assess the distribution of fatness ratings across the frames for each subject identity separately. In the first step, cumulative Gaussian functions were fitted to the fat and thin ends of the continua separately (excluding the original image), and the values and 95% confidence intervals for the best fitting curve parameters were compared, to ensure that the ratings for each end were symmetrical (i.e., that one step at the thin end was perceptually equivalent in magnitude to one step at the fat end of the transform). For three male and three female identities, functions fitting the fat and the thin ends of the data set differed significantly. These identities were not used in the main experiment.

A second Gaussian function was then fitted to the whole of the continuum for each subject identity separately. The point at which the line crossed the middle of the perceptual rating scale was identified as the perceived “normal” fatness. The transformations in PsychoMorph were then rerun using the “normal” point attained from the Prism data as the new “zero.”

In instances where the “normal” point for the identity was greater than ±45% of the original transform (14 females, 11 males), that identity was not used for the adaptation phase. 20 female and 20 male subject identities were used in the adaptation phase. Images used for fat adaptation were 10 kg of apparent fat mass above the “normal” point for the identity, and images for the thin adaptation were 10 kg of apparent fat mass below the normal point for the identity.

### Procedure

#### Baseline Phase

A method of adjustment app was coded in Matlab with the Psychophysics Toolbox, which allowed observers to cycle through the frames of transforms by moving the mouse horizontally, giving the appearance that observers were manipulating the adiposity of the subject identities. Observers were presented with 10 same sex subject identities (twice each) and were informed that moving the mouse horizontally would change the appearance of the body (they were not told it was a fat transform) and were instructed to “make each body look as normal as possible” before clicking to save the data and move onto the next identity. The mean fat mass chosen as “normal” was defined as the baseline PSN for the observer. Identities were presented in random order, and the starting frame was randomized.

#### Adaptation

In the adaptation phase, observers viewed 20 either high fat or low fat own-sex images, presented twice each in a random order. While viewing these images, they were asked to either rate the fatness (fatness attention condition) or the sex typicality (sex typicality attention condition) of the image using the whole range of a 13-point Likert scale. In the fatness attention condition, the scale ranged from very thin (1) to very fat (13). In the sex typicality attention condition, the scale ranged from very feminine (1) to very masculine (13). Immediately following this, observers completed another PSN method of adjustment task consisting of 10 same sex subject identities twice each in a randomized order. Observers were again asked to “make the body as normal as possible” and by moving the mouse from left to right they were able to adjust the bodies to establish their adapted PSN. After every three PSN trials, a “top up” adaptation stimulus was presented, for which the observer was asked to make the same Likert scale rating as during the initial adaptation phase. No set time limits were used for trials, but participants took approximately 5 s per adaptation or “top up” trial and 11 s per PSN trial. Thus, the adaptation phase lasted approximately 3.5 min and, during the post-adaptation phase, participants experienced a 5 s “top up” trial for every approximately 15 s of PSN trials.

## Results

### PSN Data

**FIGURE 3:**
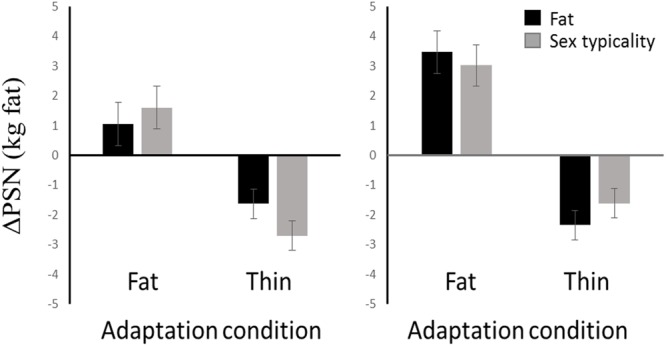
**Magnitude and direction of adaptation effects to fat and thin bodies.** Black bars show the fatness attention condition. Gray bars show the sex typicality attention condition. Female data are presented in the **(Left)** and male data in the **(Right).** Error bars show standard error of the mean. All adaptation effects are in the predicted directions.

The change in PSN (ΔPSN) between baseline and adaptation was calculated and used as the dependent variable (see **Figure 3**). Positive values represent a shift to a fatter PSN, while negative values represent a shift to a thinner PSN. The responses of one outlier in the thin adaptation condition (ΔPSN greater than 2.5 standard deviations from the thin mean), were excluded from further analysis, though the pattern of results was similar with the outlier included.

SPSS Version 22 was used for all analyses. A 2 (attention) × 2 (sex) × 2 (adiposity) between-groups ANOVA was conducted with ΔPSN as the dependent variable.

A significant main effect of adiposity was found [*F*(1,81) = 98.78, *p* < 0.001, $\eta_{p}^{2}$ = 0.55]. One samples *t*-tests confirmed that observers in the fat adaptation condition had a significantly positive ΔPSN [*M* = 2.30, *SD* = 2.52, *t(44)* = 6.11, *p* < 0.001], while the ΔPSN for observers in the thin adaptation condition was significantly negative [*M* = -2.07, *SD* = 1.66, *t(43)* = 8.28, *p* < 0.001], demonstrating aftereffects in the predicted directions (**Figure 3**).

A significant main effect of gender was found (F1,81) = 5.78, *p* = 0.018, $\eta_{p}^{2}$ = 0.07, with males’ mean ΔPSN being positive (*M* = 0.69, *SD* = 3.27), while females’ was negative (*M* = -0.42, *SD* = 2.75). The interaction between gender and adiposity was marginally significant [*F*(1,81) = 3.88, *p* = 0.052, $\eta_{p}^{2}$ = 0.05]. Independent samples *t*-tests showed that men (*M* = 3.23, *SD* = 2.06) showed a stronger aftereffect than women (*M* = 1.33, *SD* = 2.64) in the fat adaptation condition [*t*(43) = 2.71, *p* = 0.010] (though correcting for multiple comparisons made this result non-significant. No significant differences were found between the magnitude of men’s (*M* = -1.98, *SD* = 1.87) and women’s (*M* = -2.17, *SD* = 1.47) aftereffects in the thin adaptation condition [*T*(62) = 0.38, *p* = 0.707].

The interaction between adiposity and attention condition was not significant [*F*(1,81) = 0.07, *p* = 0.792, $\eta_{p}^{2}$ = 0.00], suggesting that featural attention does not affect the magnitude of the body size adaptation effect.

All other main effects and interactions were non-significant and not relevant to our hypotheses (all *F* < 2.6, all *p* > 0.11).

To test whether the magnitude of aftereffects differed depending on whether observers adapted to fat or thin stimuli, the ΔPSN data for the thin condition were multiplied by (-1), while data for the fat condition were left untransformed. Thus positive numbers represented a change in the predicted direction in both fat and thin adaptation conditions. An independent-samples *t*-test showed no significant difference in the magnitude of fat (*M* = 2.30, *SD* = 2.53) and thin (*M* = 2.07, *SD* = 1.66) aftereffects for both sexes combined, [*t*(76.27) = 0.50, *p* = 0.616]. However, since differences were seen between men and women in the magnitude of aftereffects, separate independent samples *t*-tests were performed for male and female observers. No significant difference was seen between the magnitude of fat (*M* = 1.33, *SD* = 2.64) and thin (*M* = 2.17, *SD* = 1.47) aftereffects for women [*t*(32.81) = 1.31, *p* = 0.200]. For men, the fat aftereffect (*M* = 3.23, *SD* = 2.06) was significantly larger than the thin (*M* = 1.98, *SD* = 1.87) aftereffect [*t*(43) = 2.14, *p* = 0.038]. However, the *p*-value for this effect in males is non-significant when alpha is Bonferroni adjusted for multiple comparisons.

Since the time course of body size aftereffects is not well known, we repeated the analysis on just the first five adapted PSN trials (25%) and on just the last five adapted PSN trials (25%) performed by each participant. The pattern of results did not differ from that presented above, though the interaction between gender and adiposity (marginally significant *p* = 0.52 above) was non-significant for both the first five trials (*p* = 0.139) and the last five trials (*p* = 0.636). Next, the ΔPSN data for the thin condition were multiplied by (-1), while data for the fat condition were left untransformed. Thus positive numbers represented a change in the predicted direction in both fat and thin adaptation conditions. A paired samples *t*-test showed no significant difference between the magnitude of aftereffects in the first and last five trials [*t*(71) = 0.467, *p* = 0.642]. This suggests that the “top up” trials were sufficient to prevent any significant decay of the aftereffect over the time course of the adaptation PSN trials.

### Rating Data

Two separate 2 (time) × 2 (size) mixed ANOVAs were performed on the first 10 trials (25%) and last 10 trials (25%) of the body rating data from the adaptation phase – one for the fat attention condition, and one for the sex typicality attention condition. For the sex-typicality attention condition, the main effects of size and time, and the interaction between the two were all non-significant (all *F* < 0.66, all *p* > 0.42), suggesting that adapting to fat or thin bodies did not affect the perception of the sex typicality of the bodies.

For the fat attention condition, the main effect of size was significant (F1,35) = 71.10, *p* < 0.001, $\eta_{p}^{2}$ = 0.67), showing that bodies in the thin condition were rated as thinner than the bodies in the fat condition. The main effect of time [*F*(1,35) = 1.61, *p* = 0.213, $\eta_{p}^{2}$ = 0.04] was non-significant. A significant interaction between time and size was found [*F*(1,35) = 16.60, *p* < 0.001, $\eta_{p}^{2}$ = 0.32]. Follow-up independent-samples *t*-tests showed that thin bodies were rated as thinner than fat bodies for both the first 10 trials [thin *M* = 4.37, *SD* = 1.11, fat *M* = 8.41, *SD* = 1.42, *t*(35) = 9.66, *p* < 0.001] and the last 10 trials [thin *M* = 5.47, *SD* = 1.06, fat *M* = 7.83, *SD* = 1.60, *t*(35) = 5.30, *p* < 0.001]. Paired samples *t*-tests showed that, for the thin condition, bodies were rated as less thin in the last 10 trials (*M* = 5.48, *SD* = 1.06) than in the first 10 trials [*M* = 4.37, *SD* = 1.11, *t*(18) = 3.91, *p* = 0.001]. For the fat condition, a marginal effect suggested that bodies were rated as less fat in the last 10 trials (*M* = 7.83, *SD* = 1.60) than in the first 10 trials [*M* = 8.41, *SD* = 1.42, *t*(17) = 1.92, *p* = 0.072]. This suggests that participants became adapted to the size of the bodies that they were rating over the adaptation phase, viewing the bodies as less extreme at the end of the phase than at the beginning.

## Discussion

We report the results of an experiment in which observers’ featural attention was manipulated by asking them to rate either the fatness (fatness attention condition) or sex typicality (sex typicality attention condition) of fat or thin adaptation stimuli, and measured the resultant change in PSN. Aftereffects were found in the predicted directions in both attention conditions and in male and female observers, in line with previous studies (Winkler and Rhodes, 2005; Glauert et al., 2009; Hummel et al., 2012a,b). These adaptation effects were symmetrical in magnitude for female observers. A marginal interaction effect between gender and adiposity may have raised suspicions that fat and thin adaptation aftereffects may not be symmetrical in magnitude for male observers, who showed a larger mean PSN change after adapting to fat stimuli compared to thin stimuli. However, the *post hoc t*-tests were not significant when Bonferroni corrected, and this effect was not found when analyzing the initial five adapted trials, or the final five adapted trials. Therefore, we have no evidence to suggest that aftereffects are larger for fat or thin adaptors.

Manipulations of featural attention to the fatness or sex typicality of bodies had no discernible effect on the strength of the body size aftereffect. While both spatial attention (Yeh et al., 1996; Spivey and Spirn, 2000; Rezec et al., 2004) and object attention (Shulman, 1992; Spivey and Spirn, 2000) have been shown to affect the strength of low level aftereffects, and object attention has been shown to impact the strength of face identity aftereffects, our results suggest that featural attention does not have the same effect on body stimuli. While featural attention has been found to affect the strength of the low level motion aftereffect (Lankheet and Verstraten, 1995; Boynton et al., 2006), our results add to recent findings that featural attention does not affect the strength of adaptation aftereffects in higher level stimuli, such as faces (Davidenko et al., 2016). It may be that when the different complex featural dimensions of the target object (such as fatness vs. sex typicality) are differentiated by variations in the appearance of the same local aspects of the image (such as width of hips or width of waist), attention cannot selectively modulate adaptation to the two complex featural dimensions.

Our results suggest that increasing featural attention to body size (by rating fatness vs. rating sex typicality) does not increase the size of the body size aftereffect. It may be the case, however, that our attentional manipulation was ineffective at inducing participants to attend to cues to fatness (in the fatness attention condition) and non-fatness cues (in the sex typicality attention condition), due to overlapping concepts of fatness and sex typicality. While there is an association between sex and fat mass (the healthy range for young Caucasian women is 21–33% fat, but for men it is 8–21% fat; Gallagher et al., 2000; Frankenfield et al., 2001), the variation in body shape associated with sex typicality (femininity is associated with wider hips, narrower waist, larger breasts; Furnham et al., 1998) is substantially different to the shape variation associated with body fat variation within sex (fatter people have wider hips, wider waist, larger breasts, larger stomach; Cornelissen et al., 2009). This suggests that different body cues are important when judging fatness and sex typicality, and that the two are unlikely to be conflated. Further, the analysis of the rating data from the adaptation phase showed that, while exposure to fat or thin bodies was associated with a reduction in how fat or thin the bodies were perceived to be across the adaptation phase, this exposure to fat or thin bodies was not associated with a change in the perceived sex typicality of the bodies across the adaptation phase. This suggests that fatness and sex typicality may be processed by separate channels in the brain.

Our results also have a number of implications for the techniques used in research into body aftereffects. The method of adjustment task was successful in measuring the PSN for body size, allowing us to successfully detect aftereffects in all conditions. This technique provides a quicker, more efficient alternative to the staircase tasks that are typically employed in research into aftereffects (e.g., Hummel et al., 2012a,b; Gwinn and Brooks, 2013, 2015a,b; Brooks et al., 2015). We also used a more realistic technique for manipulating the apparent fatness of bodies than has been used in previous body adaptation studies. By manipulating images of real people along an empirically derived body fat axis, our stimuli give a more realistic depiction of how people’s bodies change as their body fat levels change (Brierley et al., 2016; Sturman et al., submitted). We suggest that this technique provides a more ecologically valid method for producing stimuli than those that rely on simple geometric transforms, such as widening the bodies or by graphics manipulations that simulate an increase of the surface area.

## Conclusion

In a visual adaptation paradigm, we have demonstrated the utility of a more ecologically valid technique for manipulating stimuli along a biologically relevant, empirically derived body fat axis (Brierley et al., 2016; Sturman et al., submitted), and established a novel method of adjustment task to quickly and efficiently measure PSNs (Sturman et al., submitted). By using these techniques, we have detected body size aftereffects in the predicted directions, in men and women observing fat and thin adaptation stimuli. Featural attention toward body fatness (vs. sex typicality) was not found to affect the strength of the aftereffect, suggesting that object attention is sufficient to produce the effect. Indeed, passive viewing of high or low fat bodies has been reported to induce body size adaptation aftereffects (Brooks et al., 2016).

## Author Contributions

Conception of the project: IS, CB, JM, RS, and KB. Design of the project: IS, CB, JM, RS, and KB. Acquisition of data: IS, CB, and KB. Interpretation of data: IS, CB, JM, RS, and KB. Drafting of manuscript: IS, CB, JM, RS, and KB. Final approval of manuscript: IS, CB, JM, RS, and KB. Agreement to be accountable for the work: IS, CB, JM, RS, and KB.

## Conflict of Interest Statement

The authors declare that the research was conducted in the absence of any commercial or financial relationships that could be construed as a potential conflict of interest.
